# Clonal Structure, Seed Set, and Self-Pollination Rate in Mass-Flowering Bamboo Species during Off-Year Flowering Events

**DOI:** 10.1371/journal.pone.0105051

**Published:** 2014-08-12

**Authors:** Inoue Mizuki, Ayaka Sato, Ayumi Matsuo, Yoshihisa Suyama, Jun-Ichirou Suzuki, Akifumi Makita

**Affiliations:** 1 Laboratory of Forest Science, Faculty of Bioresource Sciences, Akita Prefectural University, Akita, Japan; 2 Department of Biological Sciences, Tokyo Metropolitan University, Tokyo, Japan; 3 Graduate School of Agricultural Science, Tohoku University, Miyagi, Japan; East China Normal University, China

## Abstract

Bamboos are typical examples of highly synchronized semelparous species. Their mass-flowering events occur at supra-annual intervals but they sometimes flower on a small scale in off-years. If some bamboo ramets (culms) of a genet flower and die in off-years, whereas other culms of the same genet do not flower synchronously, the genet can still survive blooming in an off-year and could participate in the next mass-flowering event. At genet level, the effect might be similar to that achieved by synchronously reproducing iteroparous plants. In addition, if multiple genets flower simultaneously in off-years, cross-pollination will be promoted. However, it is not known whether all the culms in a genet flower synchronously and whether multiple genets flower in off-years. We determined the clonal structure of three temperate dwarf bamboo species, i.e., *Sasa senanensis*, *S. kurilensis*, and *S. palmata*, at 24 off-year flowering sites and the surrounding areas in northern Japan using seven microsatellite markers. We also estimated seed set at seven of the sites and self-pollination rates at five sites to determine off-year reproductive success. Next, we investigated whether seed sets at the culm level were related to flowering area and/or number of flowering genets, using generalized linear mixed-effect models (GLMMs). Multiple genets flowered at 9/24 flowering sites. We found that 40/96 of the genets identified had some flowering culms. Non-flowering culms were present in 24/40 flowering genets. Seed set was in the range 2.2%–12.5% and the self-pollination rate was 96.3%. In the best GLMM, seed set increased with flowering area. Seeds were produced in off-years, but cross-pollination was rare in off-years. We suggest that some dwarf bamboos may exhibit iteroparity or imperfectly synchronized semelparity at the genet level, a characteristic similar to that of other reproductively synchronous plants. We also found synchronous flowering of a few genets even in off-years.

## Introduction

Reproductive synchrony among individuals in a population at supra-annual intervals is a widespread phenomenon [1]. Cicadas (Cicadidae) [2], *Quercus* species [3], and bamboos (Bambuseae) [4] are well known examples of this phenomenon. The major adaptive significance of the synchronous periodicity in these species is believed to be successful predator avoidance and predator satiation [5], [6], as well as avoiding the problems of restricted mating opportunities or pollen shortages due to low adult population density [7], [8]. In semelparous species, individuals that reproduce in off-years reduce their lifetime reproductive fitness and are quickly eliminated from the population. In addition, in densely distributed sessile species, even if offspring were produced in off-years, they would not be able to survive beneath their parents because of competition with the parents [9]. However, in iteroparous species, individuals that reproduce in off-years still have other opportunities to produce offspring in future attempts, even if they are not successful in the off-years. Although many plants with synchronous periodicity are iteroparous, some highly synchronized semelparous plants exist, including a Lamiaceae species, *Plectranthus insignis*, some bamboo species and the genus *Strobilanthes* [9], [10].

Bamboos can sustain themselves for several decades by means of a rhizomatous growth habit [4], and each genet has multiple ramets called “culms” [11]. Many culms flower synchronously before dying on a large scale (e.g. 60 ha in Japan [12]; over a distance of approximately 120 km in Argentina and Chile [13]; over a distance of approximately 30 km in the Amazon Basin [14], and new populations are established from seeds [12], [14], [15]. However, flowering events that occur randomly, non-gregariously, and outside mass-flowering events (i.e., off-year flowering events) have also been observed in bamboos [4], [16], [17], [18] and this phenomenon is known as sporadic flowering [11].

Although the death of flowering culms after off-year flowering has been observed in bamboo [19], it has never been ascertained whether the genets that flowered in off-years were excluded from the bamboo population. Clarification is required as to whether all the culms in a genet flower synchronously in an off-year. If some culms of a genet flower and die in off-years, whereas other culms of the same genet do not flower, the genet would still survive despite blooming in off-years. In such a scenario, at the genet level, bamboo might resemble iteroparous synchronously reproductive plants, rather than highly synchronized semelparous plants. Studies on the clonal structures of off-year flowering sites are limited in number. Off-year flowering events have only been studied in two bamboo species [19], [20]. There is also a need to determine, by surveying a number of sites, whether multiple genets flower synchronously, even in off-years (but see [19], [20]); if this is the case, cross-pollination may occur.

A few studies have supported the prediction that reproductive success in off-years seldom occurs in bamboo [19], [21]. However, other studies have reported that over 10% of seeds set even in off-years [16], [22]. It is important to identify the factors that cause these differences when considering whether it is possible to recruit from seeds after off-year flowering events. Because of the probable disadvantage of off-year flowering, seed set might be influenced by flowering area, which is correlated with the number of flowering culms and/or predation rate. Generally, genetic factors strongly affect the seed set that lack self-compatibility or have imperfect self-compatibility mechanisms [23]. Therefore we need to consider effects of genetic factors, although some bamboos may have at least some degree of self-compatibility [22], [24].

In this study, we determined the clonal structures of three temperate dwarf bamboo species: *S. senanensis* (Franch & Sav.) Rehder, *S. kurilensis* (Rupr.) Makino & Shibata, and *S. palmata* (Marlic) Nakai at 24 off-year flowering sites and their surrounding areas, in northern Japan by using seven microsatellite markers to clarify: 1) whether all the ramets in a genet flowered synchronously, and 2) whether multiple genets flowered in an off-year. To investigate reproductive success in off-years, we also recorded 1) how many seeds set at seven of the 24 sites, 2) how many seeds were self-pollinated at five of these seven sites, and 3) using generalized linear mixed-effect models (GLMMs), whether the seed set at culm level was related to the flowering area and the number of flowering genets.

## Materials and Methods

### Ethics Statement

**Figure 1:**
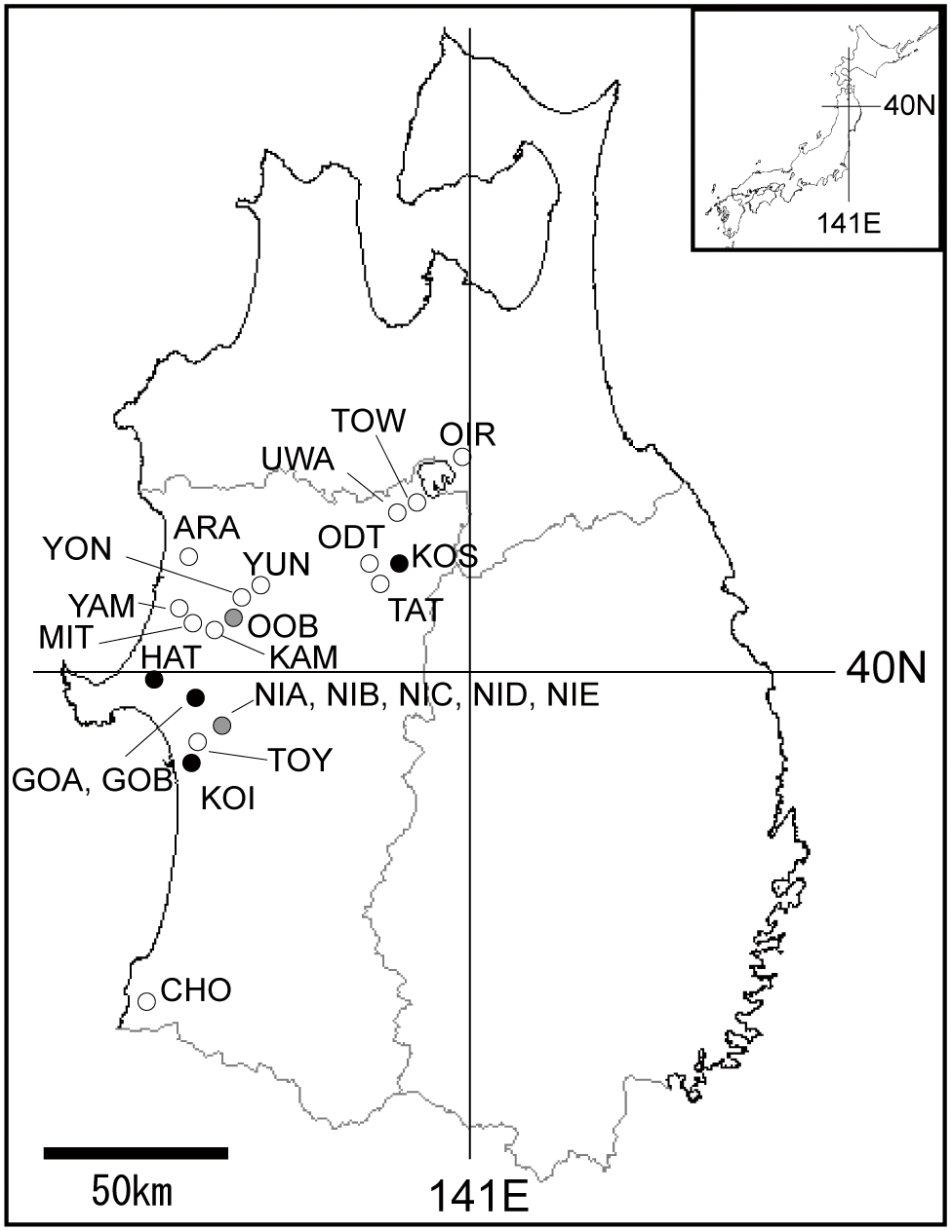
Study sites. Open circles: sites used only to determine clonal structure; gray circles: sites used to determine clonal structure and the seed set; black circles: sites used to determine clonal structure, seed set, and self-pollination rate. Some sites were too close to separate in the figure, so they are shown together.

We were given permission to survey bamboo at the GOA and GOB sites (Fig. 1) by the park management office in Gojyome Environment and Cultural village. No specific permissions were required for the other sites, or for the subsequent field studies. The bamboos we sampled were not protected or endangered.

### Species and Study Site

*Sasa* species are temperate dwarf bamboos that are widely distributed in Japan [12]. They often dominate the understory of forests, inhibiting the establishment of tree seedlings [25]. They have a leptomorph rhizome system [11] characterized by robust perennial rhizomes that are horizontal, elongated, and slender, with a single lateral bud at every node [26]. Culms, derived from some of the axillary buds on the rhizomes, grow up to 0.5–3.0 m long and live for at least 2–6 years [27], [28]. Culms from the same genet can sometimes spread up to 300 m (*S. senanensis*) [29]. The mass-flowering area of *S. kurilensis* has been reported to be over 60 ha [12]. The mass-flowering interval is estimated to be over 100 years [4], and flowering culms die after one flowering event, regardless of whether it takes place in an off-year [19] or a mass-flowering year [30].

In the Akita and Aomori Prefectures of northern Japan, we selected 24 study sites where most culms of dwarf bamboos flowered on a small scale in 2008 and 2009 (Fig. 1, Table S1). Flowering was observed from May to July, and most of the flowered culms had withered by August and September. We did not include sites where there were only a few scattered flowering culms. It was difficult to precisely identify off-year and mass-flowering events; we defined off-year flowering sites as those covering <3 ha, according to a previous definition of sporadic flowering [18]. The non-flowering sites were larger than the flowering sites, and we ensured that no other bamboos within at least a 1 km radius of these sites flowered. We also ensured that bamboos near these sites did not flower the following year. We estimated flowering area based on ellipses, by measuring the lengths of the major and minor axes of the flowering sites, and we identified the three dwarf bamboo species to be investigated at these study sites as *S. senanensis*, *S. kurilensis*, and *S. palmata*. Self-compatibility has been reported in *S. senanensis* [22], but has not been investigated in the other two species.

### Sampling

To investigate clonal structures at each site, in most cases we randomly sampled the leaves of eight flowering culms from the flowering area and those of eight non-flowering culms from the surrounding flowering area. However, where the flowering sites were large or had complex shapes other than ellipses, we sampled >15 leaves per site (Table 1). The leaves were stored at −30°C prior to DNA extraction for microsatellite analysis.

**Table 1:** Site characteristics and clonal structure at the off-year flowering sites.

				#ramets		#genets		
Species	Site	Year	Size (m^2^)	Fl.	Non-fl.	Fl.	Mix	Non-fl.
*Sasa kurilensis*	CHO	2009	130	11	11	0	1	3
	NIE	2009	131	8	8	0	1	2
	NIA	2009	169	8	8	0	1	2
	MIT	2009	201	8	8	0	2	2
	TOW	2009	447	10	8	1	1	6
	NIC	2009	698	14	10	0	1	2
	NID	2009	1790	28	16	0	1	4
*Sasa palmata*	ODT	2008	12	8	6	3	1	1
	YUN	2008	35	8	8	0	1	2
	UWA	2009	66	8	8	2	1	1
	GOA	2009	90	8	9	3	1	4
	OOB	2009	114	8	8	0	1	1
	GOB	2009	234	8	8	0	1	2
	NIB	2009	431	8	8	0	1	1
*Sasa senanensis*	YAM	2008	75	8	8	0	1	1
	ARA	2008	140	8	7	1	0	1
	TAT	2009	261	8	8	0	2	1
	KAM	2008	267	8	8	1	1	3
	TOY	2009	352	8	8	0	1	3
	YON	2009	365	10	9	0	1	1
	HAT	2009	1755	8	8	1	1	3
	OIR	2008	427	8	8	0	1	1
	KOS	2009	645	8	18	3	1	6
	KOI	2009	947	8	8	1	0	2
Total				225	214	16	24	56

Fl: flowering, Non-fl: non-flowering. Mix: both flowering and non-flowering culms were found. At sites ODT and ARA, some of the non-flowering leaves were lost in the field, so only six and seven samples, respectively, were obtained from these sites.

**Figure 2:**
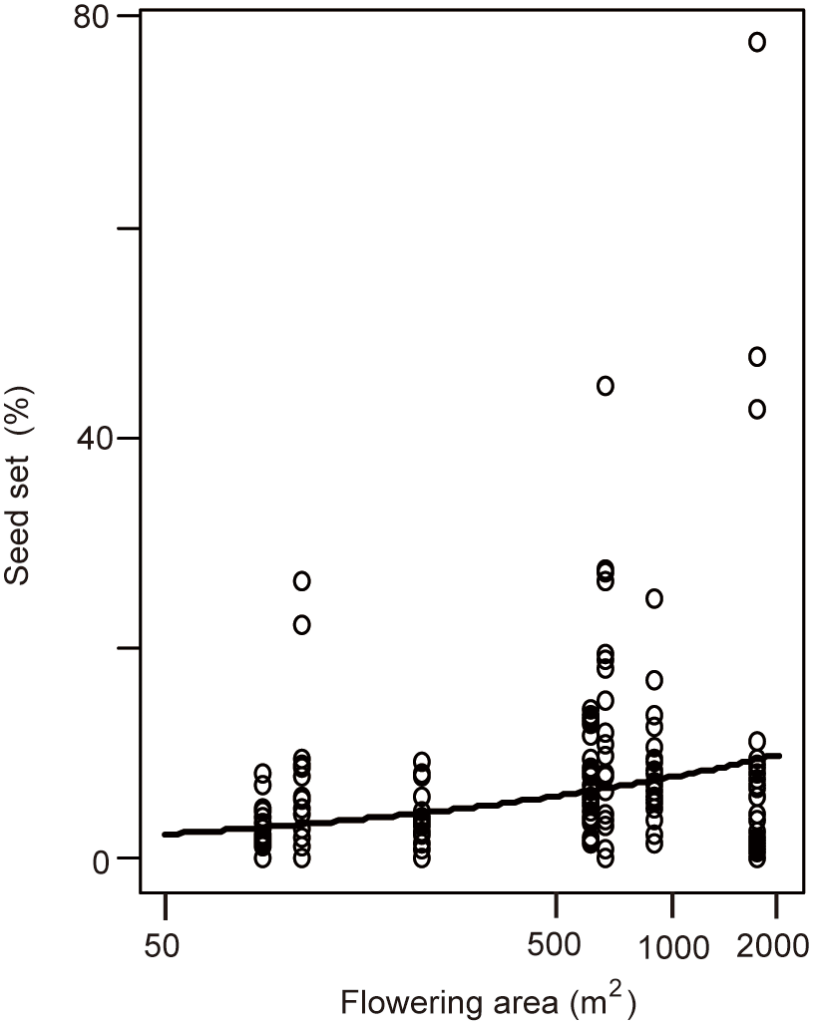
Seed set and flowering areas for the seven sites. The fitted values (solid line) are shown for the best binomial generalized linear mixed-effect model using the seven sites. Each open circle indicates the value for a single culm.

In 2009, to estimate the extent of seed set after fertilization, 30 spikes at six sites (NIC, GOA, OOB, GOB, KOS, and KOI, Fig. 1) were covered with mesh bags to prevent the loss of seeds between June and July (Fig. 2). Because the flowering area at HAT contained a road, 30 spikes were covered with mesh bags on each side of the road. After the seeds had matured, we collected the spikes that had been covered with mesh bags, although some were missing. The number of florets and the number of sound seeds were counted to estimate the seed set. Insects laid their eggs on the spikes when they flowered, so predation by insects after fertilization was not prevented by the mesh bags. Levels of seed set in this study were therefore affected by insect predation in addition to the quantity and quality of pollen.

Eighty seeds (from four sites, three to eight culms per site, and two to eight seeds per culm) were soaked in water overnight at room temperature. The seeds were then cut open and the embryos were sampled for microsatellite analysis, to estimate self-pollination rates. In addition, we sampled leaves from the culms from which seeds had been collected to identify their maternal genets.

### Microsatellite Analysis

Total genomic DNA was extracted from 50–100 mg of leaves and embryos using the CTAB (cetyltrimethylammonium bromide) method described by Murray and Thompson [31]. We performed microsatellite amplification using PCR according to the methods of Kitamura and Kawahara [20]. Genetic analysis was based on seven markers for microsatellite loci: Sasa223, Sasa500, Sasa718, and Sasa946, which have previously been characterized for *S. kurilensis* [32], and BWSS-4, BWSS-7, and BWSS-8, which had been characterized for *S. senanensis* [33]. The number of microsatellite loci used (seven) in this study was within the range used in previous studies (five to nine loci; [20], [34]) that have investigated the clonal structures of *Sasa* populations.

We used GenClone 2.0 [35] to discriminate between the genets. To ensure that the resolving power of the markers used was sufficient to distinguish genets, we calculated the probability that the observed number of identical genotypes encountered in a sample originated from sexual reproduction (*P*_sex_) [36]. The allele frequency within each population should be used to calculate *P*_sex_ [36], but we used the allele frequencies derived from all of the culms for each species instead of those derived from each population because we found only 2–10 multilocus genotypes in each population. Brzyski and Culley [37] also used the allele frequency derived from all ramets of multiple *Spiraea virginiana* populations. If a species has a high level of clonality, there is no option other than to use the allele frequencies across populations. Very low *P*_sex_ values (<1.0×10^−13^) for all genotypes were found on the basis of multiple culms in this study, so the seven microsatellite marker pairs we used were sufficient to discriminate between the genets of the three dwarf bamboo species. The total numbers of alleles we identified using BWSS-4, BWSS-7, BWSS-8, Sasa223, Sasa500, Sasa718, and Sasa946 in this study were 12, 11, 10, 27, 4, 22, and 12, respectively. All the alleles in the seeds were also found in the leaves. In contrast, using eight microsatellite loci, which included some of the same loci that we used (Sasa223, Sasa500, Sasa718, and Sasa946), Kitamura and Kawahara [20] found 2–15 alleles per locus (mean = 5.1) in 23 remote clumps of *S. cernua* collected from a 150-ha forest. We therefore assumed that multiple culms with identical multilocus genotypes were members of the same genet.

At four of the 24 sites (GOB, HAT, KOS, and KOI), we identified the pollination mode (self-pollinated or out-crossed) of the seeds using the seven loci described above. If the set of alleles at the seven loci was a subset of the maternal culm alleles, we assumed that they were self-pollinated seeds. However, if the seeds had a different allele from any in the maternal culms, we assumed them to be out-crossed seeds. We only found two to ten multilocus genotypes in each population, so we did not use the MLTR program [38], which requires the allele frequency in each population in order to estimate out-crossing rates.

### Statistical Analysis

The seed sets at culm level were estimated using GLMMs (with a binomial distribution and a logit link), with the flowering area and the number of flowering genets as independent variables. The flowering area was sometimes affected in a nonlinear manner, so we log-transformed it for the model. We did not consider the “genet” as a random effect. Bolker et al. [39] recommended that at least five to six random effect levels per random effect should be included when using GLMMs. However, we only found one or a few flowering genets per site (see Results). We therefore only included the factor ‘site’ as a random effect. We did not consider the interaction between the flowering area size and the number of flowering genets because we had insufficient data to construct complex models. The best model was selected on the basis of Akaike Information Criterion (AIC [40]). The statistical analyses were performed using R 3.0.1 [41].

## Results

### Genet Identification and Clonal Structure of the Off-year Flowering Sites

We detected 96 multilocus genotypes in 439 culms across the 24 sites (Table S2), based on seven microsatellite loci. At each site i.e. in the off-year flowering area and its surroundings, we found between two and ten genets (Table 1). A single genet flowered at 62.5% of the flowering sites (15 of 24 sites). The maximum number of genets that flowered synchronously at any one site was four (ODT, GOA, and KOS). Of the 96 genets, 40 had some flowering culms and 60.0% of these (24 of the 40 genets) also had non-flowering culms.

### Seed Set and Self-Pollination Rate

Seed set at the seven sites ranged from 2.2–12.5% (Table 2). The difference between the AIC of the model that included both flowering area and the number of flowering genets as independent variables, and that of the model that included only flowering area, was <2 (Table 3), but the latter model was simpler than the former. We therefore, selected the model that included only flowering area as an independent variable. We found that seed set increased with an increase in flowering area (Fig. 2).

**Table 2:** Seed set and self-pollination ratios for the dwarf bamboo species at the off-year flowering sites.

Species	Site	Spikesanalyzed	Florets per spikesMean (SD)	Seeds per spikesMean (SD)	Seedset (%)	SeedsAnalyzed	Self-pollinatedseeds	Self-pollinationratio (%)
*Sasa kurilensis*	NIC	20	183.2 (56.6)	22.9 (17.0)	12.5	-	-	-
*Sasa palmata*	GOA	26	77.9 (26.4)	1.7 (1.8)	2.2	-	-	-
	OOB	24	53.8 (27.6)	2.3 (2.4)	4.2	-	-	-
	GOB	28	103.0 (36.9)	3.5 (2.3)	3.4	14	12	85.7
*Sasa senanensis*	HAT	43	106.6 (57.7)	5.6 (11.7)	5.6	23	22	95.7
	KOS	27	269.4 (107.9)	19.5 (12.7)	7.4	21	21	100
	KOI	26	113.8 (45.6)	9.3 (6.8)	8.2	22	22	100

**Table 3:** Candidate models to explain variation in seed set.

Rank	AIC	Δ AIC	Model combination
1	1412.9	0.0	log(Area)
2	1414.1	1.2	log(Area)+*G*
3	1417.4	4.5	*G*

Area and *G* represent the flowering area and the number of flowering genets, respectively.

In order to estimate self-pollination rates, we compared the genotypes of all the maternal culms whose seeds were analyzed at the four off-year flowering sites. And we ensured that these genotypes were already present in the analysis of the clonal structure of the four off-year flowering sites. Analyzing 80 seeds of these maternal culms, all alleles of 77 seeds detected at the seven microsatellite loci were also found in the maternal culms (Table 2, Table S3). We therefore assumed these seeds to be the result of self-pollination. However, in the other three seeds at least one allele among the seven loci was different from those in the maternal culms. We assumed that these seeds had been produced by out-crossing. One seed at the HAT site had alleles at three loci (Sasa223, Sasa500, and Sasa946) that differed from the alleles of the maternal culm. The alleles at Sasa500 and Sasa946 were found in other, non-flowering, genets at the same site, but the Sasa223 allele was not found in any genet at the HAT site. The other two seeds that we assumed to result from out-crossing were found at the GOB site. One of these had an allele different from that of the maternal culm at the BWSS7 locus. The other had two such alleles at two loci (Sasa500 and Sasa946). These three alleles were found in the non-flowering genets at the GOB site.

## Discussion

### Clonal Structure at the Off-year Flowering Sites

The flowering culms of bamboo are destined to die, so 16 (one genet in *S. kurilensis*, eight in *S. palmata*, and seven in *S. senanensis*, Table 1) out of the 40 flowering genets that we identified would be lost from the populations after blooming in off-years because they have only flowering culms. If this was the only pattern of off-year flowering events occurring in these species, it could be disregarded as a factor in the evolution of synchronized semelparity, since the genets concerned could not contribute to subsequent mass-flowering events. However, we found that 24 out of the 40 flowering genets (eight genets in *S. kurilensis*, seven in *S. palmata*, and nine in *S. senanensis*) had non-flowering culms, in addition to flowering culms. We did not detect any flowering and/or the death of bamboos in the adjacent areas after the off-year flowering events (Mizuki et al. unpublished data), and the 24 genets with both flowering and non-flowering culms survived after the off-year flowering events. This suggests that these genets might be able to participate in the next mass-flowering and/or off-year flowering event. Although the culms exhibited semelparity, the three bamboo species investigated in this study should be categorized as iteroparous or imperfectly synchronized semelparous, rather than strictly semelparous, at the genet level. This pattern resembles that in other iteroparous synchronized species, such as *Chionochloa* species, which produce no flowers in some years [42], or *Quercus* species [3].

The detection of asynchrony within a genet of bamboo has never been confirmed [19]. Previous studies [27], [43], [44] have reported high reproductive synchrony within a genet, or even within a population of bamboo species. For example, culms that grew from rhizomes from a *Phyllostachys pubescens* seed were transplanted, 40 years after the germination of the seed, to two sites that were located more than 500 km apart. The culms of both sites flowered at the same time, 66 years after germination [27], [43]. Transplants of other bamboo species, such as *S. kurilensis* var. *jotanii*, have also flowered at the same mass-flowering time as their parental populations, although in one case they were located more than 300 km north of these populations [44]. These previous studies show that the biological clocks of bamboos may be resistant to environmental variation (but see [9]). The proximate factors that cause off-year flowering events in bamboos should be investigated in future studies.

We found that 15 (62.5%) out of the 24 off-year flowering sites had only one flowering genet. Previously, this pattern was found in a site with *S. cernua* [20], and a site with *S. pubiculmis* [19], both of which exhibited off-year flowering. However, we also found that multiple genets flowered synchronously (at 9 of 24 sites, 37.5%) in all three species. The maximum number of genets that flowered synchronously at the same site was four (ODT, GOA, and KOS). This is far fewer than typically flower in a mass-flowering year (for example, nearly 200 flowering genets have been observed in *S. veitchii* var. *hirsuta*; Matsuo et al. unpublished data). Although the chance of out-crossing during off-year flowering events would not be zero, it would still be small.

### Seed Set and Self-Pollination Rate

The seed set at the culm level ranged from 2.2–12.5% across the seven sites, this is within the usual range for off-year flowering populations of bamboo. For example, in off-years, the seed set of *S. senanensis* ranged from 1–24%, with a mean of 9.1% [16], those of *S. nipponica* and *S. senanensis* were 12.8% and 10.6%, respectively [22], and those of *Dendrocalamus giganteus* ranged from 0–9% [21]. These values were lower than those in mass-flowering populations of *S. kurilensis* var. *jotanii* (20.1–26.0%, [30]), or in *Sinarundinaria fangiana* (34%, [45]), but similar to those of mass-flowering populations of *Sasa tsuboiana* (6.0–20.8%, [46]) and *Sasa veitchii* var. *hirsuta* (6.4% and 7.5%, [47]). Seed set values in our study were higher than those of *S. fangiana* (0.6%) in the year after a mass-flowering event [45].

We found that seed set increased with flowering area. Seed set can be affected by four main factors: predation, pollen and resource availability, and genetic factors [23]. Flowering area is relevant to the first two of these factors: predation and pollen availability. A larger flowering area would reduce the predation rate [5], [48] and lead to a larger quantity of pollen being released from the many flowering culms. The number of genets was not included in the best of the models used to estimate seed set. This indicates that genetic factors were not particularly important for seed sets in the off-year flowering events. Seeds were produced at sites having only a single flowering genet (NIC, OOB, GOB, and KOI), and they were highly self-pollinated, indicating that these bamboos are self-compatible or imperfectly self-incompatible; self-compatibility has previously been reported for *S. senanensis* [22].

Regardless of the number of flowering genets, seeds produced in the off-years showed extremely high self-pollination rates (96.3%, 77 of 80 seeds) compared with those of *S. veitchii* var. *hirsuta* in mass-flowering events (16%, Matsuo et al., unpublished data). This finding is consistent with an analysis of off-year flowering sites for *S. cernua* [24]. In the current study, only three seeds were identified as out-crossed (Table 2). The two seeds collected at GOB would have resulted from out-crossing within the population, because the alleles which differed from those of their maternal culms were found in the leaves of other, apparently non-flowering, genets at the site, which suggests that some culms of the “non-flowering” genets had flowered but were not identified as such in our sampling. We may have underestimated the number of flowering genets at the off-year flowering sites because of the small number of culms sampled in our study. At the HAT site, the unique alleles at Sasa223, which were found in an out-crossed seed, were not present in the leaves and seeds of any of the other genets in the population. This seed may therefore have resulted from out-crossing among local populations, although we did not find any off-year flowering sites near HAT.

Future studies should investigate whether genets that flowered in the off-year will flower in other off-years, and in the next mass-flowering year, and whether the genets that did not flower in the off-year will flower in other off-years. Long-term observations are essential for estimating the lifetime reproductive fitness of bamboo at the genet level, as well as in understanding the evolution of reproductive synchrony in plants.

## Supporting Information

Table S1 Locations of the off-year flowering sites.
(DOC)

Table S2 Summary data of culm samples.
(XLSX)

Table S3 Summary data of seed samples and their maternal culms.
(XLSX)
