# Supplementary material for: Clonal Structure, Seed Set, and Self-Pollination Rate in Mass-Flowering Bamboo Species during Off-Year Flowering Events
Source: PLoS One. 2014 Aug 12;9(8):e105051. doi: 10.1371/journal.pone.0105051 (PMC4130643; doi:10.1371/journal.pone.0105051)
Supplement: Table S1 — Locations of the off-year flowering sites. (DOC) [file pone.0105051.s001.doc]

**Table S1** Locations of the off-year flowering sites.

| Species | Site | *N* | *E* |
| --- | --- | --- | --- |
| *Sasa kurilensis* | CHO | 39.157 | 139.909 |
|  | NIE | 39.805 | 140.263 |
|  | NIA | 39.807 | 140.225 |
|  | MIT | 40.087 | 140.146 |
|  | TOW | 40.415 | 140.845 |
|  | NIC | 39.803 | 140.232 |
|  | NID | 39.805 | 140.264 |
| *Sasa palmata* | ODT | 40.213 | 140.584 |
|  | YUN | 40.177 | 140.455 |
|  | UWA | 40.387 | 140.794 |
|  | GOA | 39.929 | 140.121 |
|  | OOB | 40.086 | 140.328 |
|  | GOB | 39.927 | 140.118 |
|  | NIB | 39.807 | 140.225 |
| *Sasa senanensis* | YAM | 40.100 | 140.085 |
|  | ARA | 40.277 | 140.070 |
|  | TAT | 40.325 | 140.732 |
|  | KAM | 40.087 | 140.330 |
|  | TOY | 39.930 | 140.152 |
|  | YON | 40.168 | 140.394 |
|  | HAT | 39.971 | 140.080 |
|  | OIR | 40.575 | 140.979 |
|  | KOS | 40.335 | 140.763 |
|  | KOI | 39.816 | 140.070 |
